# Direct observation of laser guided corona discharges

**DOI:** 10.1038/srep18681

**Published:** 2015-12-18

**Authors:** Tie-Jun Wang, Yingxia Wei, Yaoxiang Liu, Na Chen, Yonghong Liu, Jingjing Ju, Haiyi Sun, Cheng Wang, Haihe Lu, Jiansheng Liu, See Leang Chin, Ruxin Li, Zhizhan Xu

**Affiliations:** 1 State Key Laboratory of High Field Laser Physics, Shanghai Institute of Optics and Fine Mechanics, Chinese Academy of Sciences, China; 2 Centre d’Optique, Photonique et Laser (COPL) and Département de physique, de génie physique et d’optique, Université Laval, Québec, Québec G1V 0A6, Canada

## Abstract

Laser based lightning control holds a promising way to solve the problem of the long standing disaster of lightning strikes. But it is a challenging project due to insufficient understanding of the interaction between laser plasma channel and high voltage electric filed. In this work, a direct observation of laser guided corona discharge is reported. Laser filament guided streamer and leader types of corona discharges were observed. An enhanced ionization took place in the leader (filament) through the interaction with the high voltage discharging field. The fluorescence lifetime of laser filament guided corona discharge was measured to be several microseconds, which is 3 orders of magnitude longer than the fluorescence lifetime of laser filaments. This work could be advantageous towards a better understanding of laser assisted leader development in the atmosphere.

Lightning as a natural atmospheric discharge phenomenon is one of the long standing and most serious natural sources of disaster. Human activities toward the protection and control of lightning have never been stopped since Benjamin Franklin’s famous kite experiments in 1752. Although lightning rod has been widely used to protect key locations and human life, it is impossible to avoid the lightning strikes because of its passively random nature. Active controls of lightning have been proposed and/or demonstrated. Rocket-triggered lightning discharges were successfully conducted many times in several countries since 1967^1^,^2^,^3^,^4^,^5^,^6^. As a more promising and interesting method, laser based lightning control has attracted much attention^7^,^8^,^9^,^10^,^11^,^12^,^13^,^14^,^15^,^16^,^17^,^18^,^19^,^20^,^21^,^22^,^23^,^24^,^25^,^26^,^27^,^28^,^29^,^30^,^31^,^32^,^33^,^34^,^35^,^36^ due to its pollution-free and good capability of high repetition rate operation and precise control of shooting direction.

The method of laser based lightning control is based on the plasma formation through the laser interaction with air molecules. High intensity laser pulses will ionize air molecules leading to the formation of a plasma channel required to guide lightning strikes. The idea was proposed in the 1970s^7^ and investigated in the following two decades by using high energy nanosecond-duration lasers^8^,^9^,^10^. The ns-laser based lightning control was abandoned by the end of 1990s because of the discontinuous plasma channel formed through avalanche ionization process. The observation of femtosecond laser filamentation in 1995^11^ opened a new opportunity for laser based lightning control^12^. Femtosecond laser filamentation is a dynamic balance between intensity depended optical Kerr self-focusing and laser ionized plasma defocusing resulting in a long plasma channel formation, referred to as a laser filament^13^,^14^,^15^,^16^,^17^,^18^,^19^. This femtosecond laser filament with high intensity (~5 × 10^13^ W/cm^2^ in air during free propagation^20^) can be projected to a long distance in the atmosphere via the controls of initial pulse chirp, beam divergence etc. The peak density of free electrons in these plasma filaments is on the order of 10^16^ cm^−3^ 21. However, the electron density decreases by more than an order of magnitude in less than 3 nanoseconds due to recombination process^22^. It is then followed by a much slower electron attachment process^23^. As a consequence, the lifetime of filament induced fluorescence in air is only a few nanoseconds, which limits the lifetime of the long plasma channel formation with high electron density. Many studies towards understanding the mechanism of filament guided discharge and developing techniques on the extension of plasma filament length and lifetime have been carried out^22^,^23^,^24^,^25^,^26^,^27^,^28^,^29^,^30^,^31^,^32^,^33^,^34^,^35^,^36^. The lifetime of long high density plasma channel is a critical parameter for lightning control since it limits the maximum length that a laser can guide the discharge. According to the traveling speed of laser guided leader discharge, ~10^5^ m/s^30^, a lifetime of high density plasma channel up to ~ms or longer would be preferred for a ~100 meters or longer leader (filament) length in atmospheric lightning application. So far, the lifetime of high electron density could be extended from a few nanoseconds to several tens of nanoseconds^22^,^23^,^24^, which is still not long enough for atmospheric lightning applications^31^. The possibility to trigger real-scale lightning in the atmosphere by laser filament has also been demonstrated although there was no direct observation of laser guided lightning strikes^31^. The observations^31^,^34^ suggested that corona discharges are important and may have been triggered during the interaction between laser filaments and high voltage electric field. Sugiyama *et al.*^35^,^36^ reported the dynamics and kinetics of laser filaments in a strong non-uniform electric field both experimentally and theoretically. A corona discharge is, by definition, a gas discharge where the geometry confines the gas ionizing processes to a high-field ionization region around an active electrode^37^. Currently, what one knows in the thunderstorm is the following: there exist positive and negative static HV electric fields and there are a lot of corona discharge (leader) and streamer development before intra or extra cloud lightning^31^. Corona discharge plays a very important role in the leader initiation process related to lightning^38^ and also find significant applications on chemical processing^39^. Lightning electric field could be either guided to the earth^29^ or neutralized in the intra/extra thunder cloud^31^ by laser filaments as proposed. The success of both cases would strongly rely upon the propagation of corona along the plasma filament. However, a direct observation of filament guided corona discharges with high contrast ratio and a detailed investigation on the interaction between laser filament and corona discharges are still not yet fully explored.

In this work, we report on a high contrast, direct observation of laser filament guided corona discharges (FGCD). The ionization inside the plasma channel can be enhanced through the interaction of the strong laser field in the filament with the external high voltage discharging field, leading to a higher density plasma channel. The plasma density was inferred by the strength of the fluorescence emission from inside the filament. The fluorescence lifetime of this high density plasma channel was measured to be several microseconds, which was 3 orders of magnitude longer than the lifetime of pure laser filament induced fluorescence. The results would not only benefit the understanding of the interaction between plasma filaments and corona discharges, but also stick out as the first step towards laser assisted leader development in the atmosphere.

## Experimental Setup

**Figure 1:**
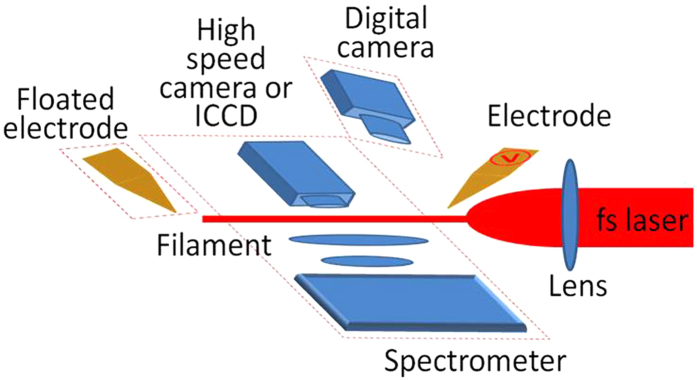
The schematic of experimental setup.

The experiments were conducted by using a 1 kHz/25 fs Ti:sapphire chirped pulse amplification system delivering pulse energy up to 10 mJ. Laser filaments were created by a plano-convex lens with a focal length of 30 cm. The schematic of experimental setup is shown in Fig. 1. All the high voltage electric field discharge experiments were performed in a home-made Faraday cage. High voltage corona discharges were generated by using a copper electrode with a 1 mm diameter tip situated at the right hand side of Fig. 1. A DC high voltage power supply with output up to 100 kV/1000 W was connected to the electrode. (The other floated electrode at the left hand side of the figure was removed during this first set of propagation experiments.) Laser filaments were created just next to the tip of the electrode at a distance of ~1 mm. Real color images were taken from the top at ~45 deg. to the vertical plane by a digital camera (Nikon D7200). Spectroscopic measurements of ionization-induced fluorescence were done by imaging the filament guided corona discharge channel into a CCD coupled spectrometer (Andor Shamrock SR-303i) from the side in the horizontal plane. An ICCD camera (Andor iStar 334T) and a high speed camera (PCO Dimax HD) were also used to capture the weak streamer structures of corona discharges and the temporal evolution of filament guided corona discharges from the side in the horizontal plane.

## Results

### Direct imaging of laser guided corona discharge

**Figure 2:**
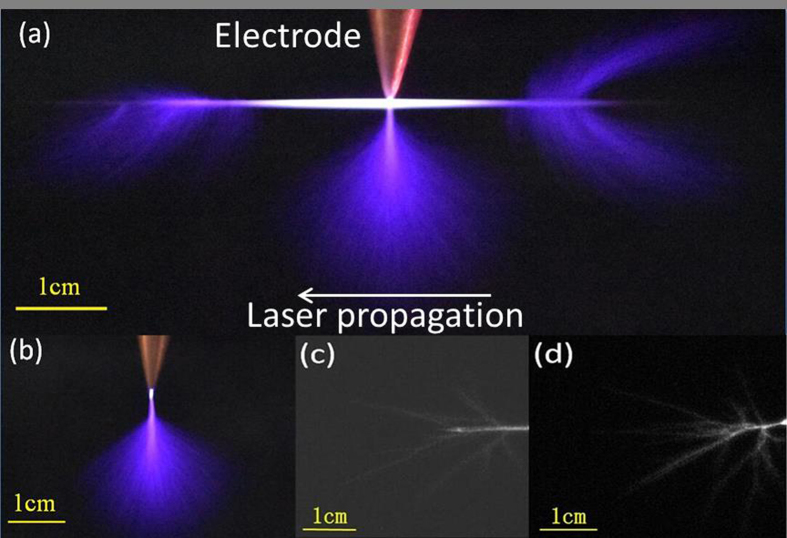
(**a**) real-color images of typical filament guided corona discharge, the filament being the white elongated horizontal region, (**b**) corona discharge without laser filament, (**c**,**d**) for the fine structures for those streamers in the forward direction of laser propagation from (**a**,**b**), respectively. The corona discharging voltage and filamenting pulse energy were 50 kV and 7.5 mJ, respectively.

Real-color image of typical filament guided corona discharge is shown in Fig. 2(a), which was taken by the digital camera. The corona discharging voltage and filamenting pulse energy were set at 50 kV and 7.5 mJ, respectively. High contrast streamer type of blue color corona discharges from both ends of the filament were observed. This indicated that the corona discharges could be guided along the laser filament in or against the propagation direction. A traditional streamer type of corona discharge radiating from the tip of the electrode can also be seen. As a comparison, the real-color image of a corona discharge without laser filament is shown in Fig. 2(b) at the same voltage of 50 kV. This blue emission comes from the ionization induced UV fluorescence of air molecules (mainly from nitrogen), which will be shown in the spectral measurements later. Using the ICCD camera, we observed in Fig. 2(c) the fine tree structures of the streamers corresponding to the real color discharge in the forward direction of laser propagation shown in Fig. 2(a). As a comparison, the tree structure of the streamers shown in Fig. 2(b) without the laser filament is shown in Fig. 2(d).

**Figure 3:**
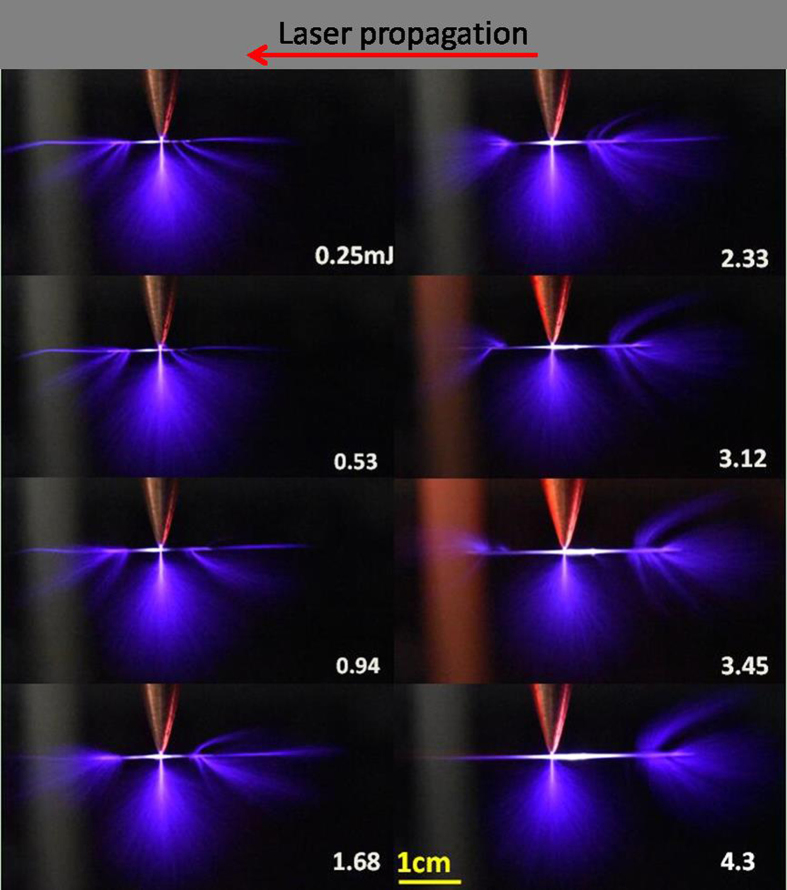
Streamer type of corona discharges propagation along laser filaments. Corona discharges were generated by applying a 50 kV high voltage on the electrode. Filament length was controlled by femtosecond laser pulse energy ranging from 0.25 mJ to 4.3 mJ.

**Figure 4:**
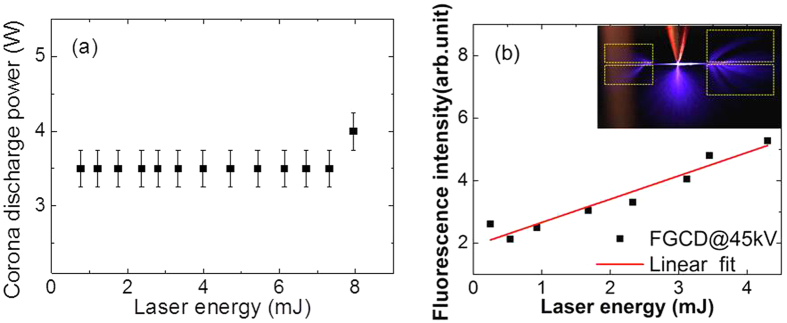
Laser pulse energy dependence of corona discharge power (a) and of FGCD induced fluorescence intensity (b). The fluorescence intensity in (**b**) was calculated by integrating the pixel intensity of the streamers in the rectangular areas as shown in the inset figure from the FGCD images in Fig. 3. Each pair of rectangles was separated so as to avoid including the direct contribution of the light coming from the on-axis filament zone.

To look into the corona discharge propagation along plasma filaments with different lengths, the corona discharging voltage was fixed at 50 kV. Laser filaments were created just next to the tip of the electrode at a distance of ~1 mm. Filamenting pulse energy was tuned from 0.25 mJ up to 4.3 mJ so as to generate different lengths of plasma filaments using a lens of 30 cm focal length. The guiding effect as a function of filamenting pulse energy is shown in Fig. 3. This guiding effect is sensitive to the position of initial corona discharges and plasma distribution of the laser filament. When a filament is short as in the cases of 0.25 mJ to 3.45 mJ, this streamer type of corona discharges can be observed at both ends of the laser filaments since the tip of the electrode is around the center of the laser filament. As the filamenting pulse energy increases, laser filaments will extend their lengths towards the focusing lens (ref. 13) (right hand side in the figure). As a consequence, more streamers are generated at the leading end (right hand side) of the filament as compared to the discharges at the trailing end. At 4.3 mJ (Fig. 3), the streamer at the trailing end (left hand side) is very weak. The total corona discharge power was monitored by the power supply to be ~3.5 W (Fig. 4(a)) while the fluorescence intensity of FGCD in Fig. 3 was obtained by integrating the pixel intensity of those streamers in the images. The integrated area is indicated as rectangles shown in the inset of Fig. 4(b). The FGCD induced fluorescence signal in Fig. 4(b) increases as the laser pulse energy increases. This indicates more streamer type of corona discharges were induced by the laser filament, although the total corona discharge power is almost constant (Fig. 4(a)) within the measurement error bars.

### Electron density enhancement in the plasma channel

**Figure 5:**
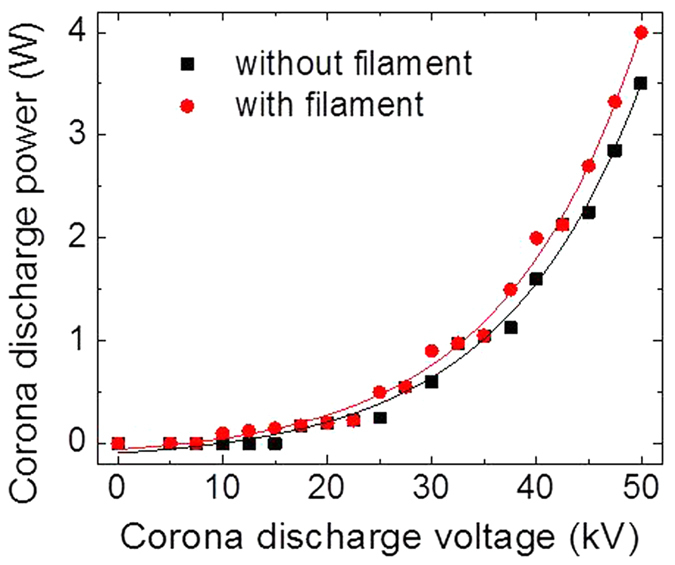
Corona discharge power as a function of supplying voltage with (red round dots) and without (black square dots) laser filaments. Red and black solid lines are exponential fittings to experimental data. Filamenting pulse energy was 7.95 mJ.

The conducting (guiding) property of a plasma channel is crucially dependent on the electron density in it. The higher the density is, the stronger the conducting ability is Fig. 5 shows a comparison of the consumed power during corona discharges with and without laser filaments. In these measurements, laser filaments were generated by focusing a 7.95 mJ laser pulse by the lens of 30 cm focal length. As the corona discharge voltage was increased, the consumed power for corona discharges exponentially increased. The corona discharges can be further increased by 10-20% through the interaction with the laser filaments, in particular, when higher voltage (in our case it was >35 kV) was applied.

**Figure 6:**
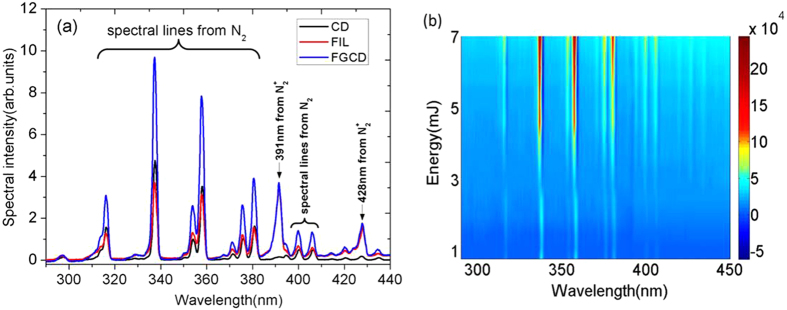
(**a**) typical fluorescence spectrum in UV (290–440 nm) emitted by corona discharge (CD), pure filamentation (FIL) and plasma channel of filament-guided corona discharge (FGCD), respectively. The voltage for corona discharge and the laser pulse energy for filamentation in the three cases were fixed at 50 kV and 7.0 mJ, respectively. (**b**) pseudocolor plot of fluorescence spectral intensity of FGCD with CD and FIL fluorescence intensity subtracted as a function of the laser pulse energy tuning range from 0.7 mJ to 7 mJ. The CD voltage was fixed at 50 kV.

This observation was confirmed by spectral measurements of the fluorescence induced by ionization processes. Fig. 6(a) depicts three typical fluorescence spectra in the UV, namely from corona discharge (CD), pure filamentation (FIL) and the plasma channel along the filament when filament guided corona discharge (FGCD) occurred. In these measurements, an identical floated electrode (left hand side in Fig. 1) was set at around 8 mm from the discharge electrode along filament propagation direction so as to easily image the discharge along the filament to the slit of the spectrometer. Adding the floated electrode would also provide us with a consistent measurement since the filament will move as the laser pulse energy is changed. A downsize imaging telescope was used to collect the fluorescence emission from the filament zone and/or the tips of the two electrodes. The voltage for the corona discharge and the laser pulse energy for filamentation in the three cases were fixed at 50 kV and 7.0 mJ, respectively. The focal length of the lens used to form a filament was 30 cm. Note that there was corona discharge spreading out from the floated electrode even without laser filament at the voltage of 50 kV. That emission was not collected in this measurement since attention here was paid on the plasma filament zone under the high voltage, which would provide key information on the ionization process, hence, plasma density (or conducting property).

The UV spectra (Fig. 6 (a)) cover the signals from the first negative band system of 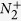
(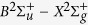 transition) and the second positive band system of 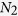
(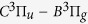 transition)^40^. It is clear that the structures of the spectra from molecular 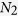 are similar in the three cases, but different at 391 nm and 428 nm which are ionic lines from 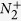 through the transitions of 
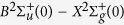
 and 
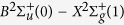
. In the case of CD, the fluorescence is mainly generated by avalanche ionization through collision process, in which the probability to populate 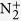 to an excited state is too low as compared to exciting neutral N_2_. While in the case of FIL, 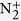 are generated by multiphoton/tunneling ionization. The laser intensity inside the laser filament is high enough to ionize N_2_ and excite 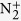 into 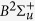 leading to ionic fluorescence^41^. Then through electron collision and dissociative recombination processes, e.g. N_2_^+^ + N_2_ => N_4_^+^; N_4_^+^ + e => N_2_^*^ + N_2_; N_2_^*^ => N_2_ + hv (337 nm, 357 nm, 380 nm etc.)^42^, neutral excited 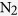 can be populated, leading to 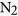 fluorescence emission (337 nm etc.). Hence, any increase in the fluorescence from N_2_ such as the 337 nm line would indicate an increase in the number of N_2_^+^; i.e. an increase in ionization. Indeed, Fig. 6(a) shows that the fluorescence from neutral N_2_ is highest in the case of FGCD. That is to say, more ionization occurred in the filament zone in FGCD; hence, the plasma density is higher in FGCD.

Further analysis was performed by the following procedure: at a fixed voltage (50 kV) of corona discharge, the fluorescence signal as a function of filamenting pulse energy ranging from 0.7 mJ to 7 mJ was measured; then to look into the interaction effect, the CD and FIL fluorescence signals were subtracted out from the FGCD signal. Fig. 6(b) depicts the pseudocolor plot of the resultant signal intensity as a function of the pulse energy. A clear positive resultant signal was obtained, which indicates that the stronger fluorescence came from the interaction. In particular, this resultant fluorescence becomes much stronger when using higher filamenting pulse energy (in our case when the energy was more than ~4 mJ). It means that under high voltage, the plasma density in the filament zone increased as the laser pulse energy increased. Among them, the increase of neutral molecular N_2_ fluorescence is much more than that from ionic 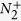. This is because photo-ionized electrons are accelerated under the external high electric field. Successive collisions would induce more ionization leading to a higher plasma density. As a consequence, more neutral excited 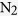 can be populated through the collisional processes mentioned above resulting in more 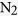 fluorescence emission. The fact that there is much less fluorescence from the excited ions 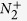 indicates that the external voltage might not be high enough to allow excitation by electron collision.

Summarizing, the observed enhancement of ionization induced neutral fluorescence indicates a higher plasma density from the interaction between the laser filament and the high electric field resulting in an efficient guiding of high voltage and the enhancement of corona discharge as observed in Figs 2.

### Lifetime extension of plasma channel

**Figure 7:**
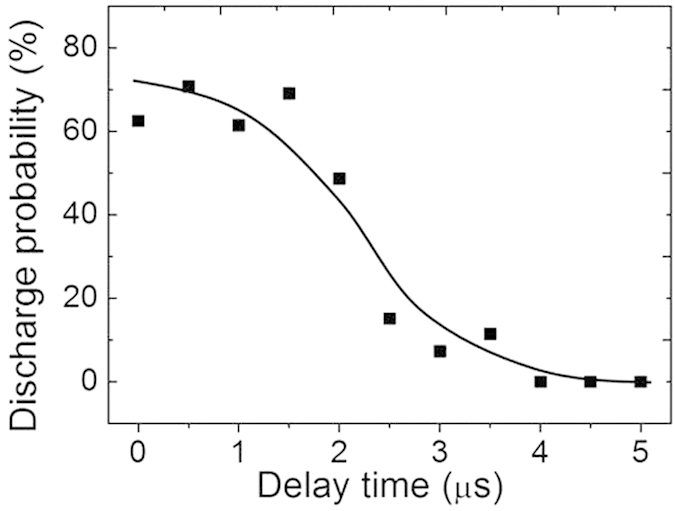
Lifetime of filament guided leader type of corona discharges. Filamenting pulse energy was 4.8 mJ. Corona discharging voltage was 50 kV.

The fluorescence lifetime of the plasma channel of filament guided corona discharges were measured by using a high speed camera. High voltage and filamenting pulse energy were fixed at 50 kV and 4.8 mJ, respectively. The length of the filament focused by a lens of 30 cm focal length was ~10 mm. In order to have a clear and stable guiding by laser filament, the tip distance of the two electrodes was set at 15 mm (see Fig. 1), a little longer than the filament length. Under this condition, filament induced corona discharge could easily bridge the gap of 15 mm between the two electrodes. Note that in these measurements, leader type of corona discharges were generated bridging the two electrodes; one electrode was floated in order to maintain the leader type of corona discharge. The exposure time of the high speed camera was 1 μs, which was triggered by the 1 kHz laser pulses. The triggering time was delayed with respect to the laser arrival time. At each delay time, hundreds of shots were recorded within a few seconds. When filament guided leader type of corona discharge bridges the two electrodes, it was counted as a successful one. The probability of successful filament guided corona discharges is shown in Fig. 7. It indicates that this guided discharge can last up to ~4 μs, which is 3 orders of magnitude longer than the nanosecond fluorescence lifetime of the plasma filament. Since the lifetime in the experiment is proportional to the amplitude of high voltage discharging field^26^, further increase of plasma lifetime can be expected by using a much stronger external electric field. This observation may solve the long standing problem of short lifetime of plasma filament for atmospheric lightning.

## Discussion

There are two types of corona discharges, namely streamer at both ends of the filament and leader along the filament path, observed in the filament guided corona discharges. The main mechanisms involved in the laser filament guided corona discharges include the photo-ionization, impact ionization, electron attachment on oxygen molecules and the detachment of these electron by various processes such as electron collision, ion collision, etc., charged particle recombination, electron diffusion etc.^23^,^29^. The high intensity inside the laser filament not only ionizes air molecules (mostly N_2_ and O_2_), but also excites molecular ions and neutrals into high lying states resulting in fluorescence emission of neutrals and ions^13^,^14^,^15^,^41^. The long low density plasma channel is the key to guide high voltage and to form the corona discharge at the two tips of the filament. The filament is firstly heated by the Joule effect after electron-ion recombination and then hydrodynamically expands outward resulting in a low pressure in the filament relaxation zone^43^,^44^. The lower pressure enhances the electrical conductivity because of less collision of electrons.

When the positive high voltage electrode was applied to the filament, the free electrons inside the filament zone would be accelerated with less collision because of the low pressure enhancing the ionization along their path. As a consequence, laser guided leader type of corona was generated. This would enhance the fluorescence. At the same time, because electrons are pushed and/or attracted to the electrode at high positive voltage, the filament would become positively charged. The two sharp ends of the filament would accumulate more positive charges giving rise to a high field. The strong electric fields at the two sharp extremities of the filament would then induce the local streamer type of corona discharges as shown in Fig. 2(a). The laser initiated electrons together with corona discharged electrons undergo impact ionization resulting in a higher plasma density. As a consequence, corona discharges are enhanced (as seen by corona discharge power measurement in Fig. 5), which is also confirmed by the spectral measurement of filament guided corona discharge (FGCD) induced fluorescence in Fig. 6.

The longer decay time of the FGCD could be understood as follows. During FGCD, photo-ionization is an ultrafast process, which occurs within the duration of the femtosecond laser pulse. The attachment of electrons onto oxygen molecules is detrimental to the lifetime of the plasma channel. When the external electric field is added along the plasma channel, the detachments of electrons from oxygen molecules will be increased and the electron density of the plasma channel will be enhanced, leading to a much longer decay time in the FGCD channel as was observed in Fig. 7^23^.

## Conclusion

Laser filament guided streamer and leader types of corona discharges were directly observed. The electron density in the plasma channel from the interaction of laser filament with high voltage discharging electric field can be enhanced under the condition of single or multiple filamentation. This plasma channel possesses the good properties of a much longer lifetime as compared to the lifetime of pure filaments. This would enhance the probability for long distance electrical guiding in the atmosphere.

## Additional Information

**How to cite this article**: Wang, T.-J. *et al.* Direct observation of laser guided corona discharges. *Sci. Rep.* **5**, 18681; doi: 10.1038/srep18681 (2015).
